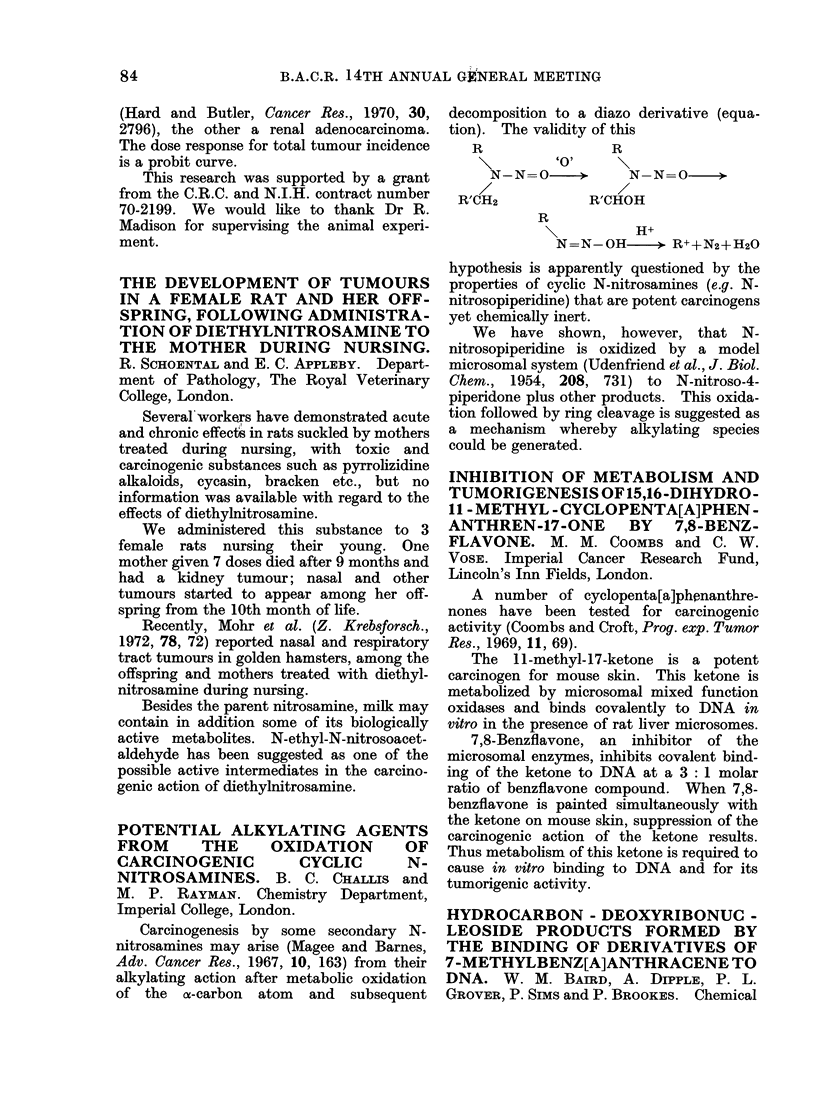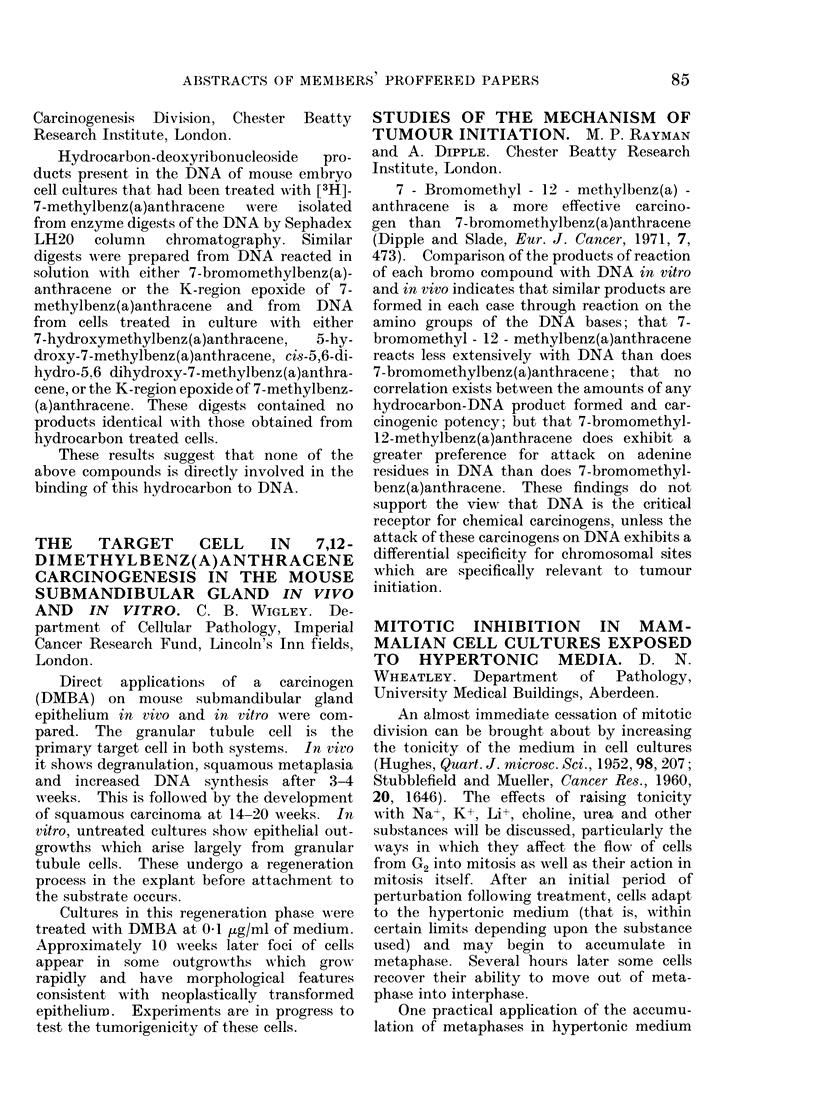# Hydrocarbon-deoxyribonucleoside products formed by the binding of derivatives of 7-methylbenz(A)anthracene to DNA.

**DOI:** 10.1038/bjc.1973.103

**Published:** 1973-07

**Authors:** W. M. Baird, A. Dipple, P. L. Grover, P. Sims, P. Brookes


### HYDROCARBON - DEOXYRIBONUCLEOSIDE PRODUCTS FORMED BY THE BINDING OF DERIVATIVES OF 7-METHYLBENZ[A]ANTHRACENE TO DNA.

W. M. Baird, A. Dipple, P. L. Grover, P. Sims and P. Brookes. Chemical

Carcinogenesis Division, Chester Beatty Research Institute, London.

Hydrocarbon-deoxyribonucleoside products present in the DNA of mouse embryo cell cultures that had been treated with [$^3$H]-7-methylbenz(a)anthracene were isolated from enzyme digests of the DNA by Sephadex LH20 column chromatography. Similar digests were prepared from DNA reacted in solution with either 7-bromomethylbenz(a)-anthracene or the K-region epoxide of 7-methylbenz(a)anthracene and from DNA from cells treated in culture with either 7-hydroxymethylbenz(a)anthracene, 5-hydroxy-7-methylbenz(a)anthracene, *cis*-5,6-dihydro-5,6 dihydroxy-7-methylbenz(a)anthracene, or the K-region epoxide of 7-methylbenz-(a)anthracene. These digests contained no products identical with those obtained from hydrocarbon treated cells.

These results suggest that none of the above compounds is directly involved in the binding of this hydrocarbon to DNA.